# Triage of HR-HPV Positive Women with Minor Cytological Abnormalities: A Comparison of mRNA Testing, HPV DNA Testing, and Repeat Cytology Using a 4-Year Follow-Up of a Population-Based Study

**DOI:** 10.1371/journal.pone.0090023

**Published:** 2014-02-26

**Authors:** Maria Persson, K. Miriam Elfström, Sophia Brismar Wendel, Elisabete Weiderpass, Sonia Andersson

**Affiliations:** 1 Division of Obstetrics and Gynecology, Department of Women’s and Children’s Health, Karolinska Institutet, Karolinska University Hospital Solna, Stockholm, Sweden; 2 Department of Medical Epidemiology and Biostatistics Karolinska Institutet, Stockholm, Sweden; 3 Cancer Registry of Norway, Oslo, Norway; 4 Department of Community Medicine, Universitetet i Tromso, Tromso, Norway; 5 Samfundet Folkhälsan, Genetic Epidemiology Group, Folkhälsan Research Center, University of Helsinki, Helsinki, Finland; The Chinese University of Hong Kong, Hong Kong

## Abstract

**Objective:**

Expression of the viral E6/E7 oncogenes of high-risk human papillomaviruses (HR-HPV) is necessary for malignant conversion and maintenance in cervical tissue. In order to determine whether HR-HPV E6/E7 mRNA testing more effectively predicts precancerous lesions and invasive cervical cancer than HR-HPV DNA testing, we aimed to compare triage using HR-HPV E6/E7 mRNA testing by APTIMA HPV Assay (APTIMA) to HPV16 DNA testing, HPV16/18 DNA testing, and repeat cytology.

**Methods:**

Liquid-based (PreservCyt) cell samples were obtained from HR-HPV-positive women diagnosed with atypical squamous cells of undetermined significance (ASCUS) and low-grade squamous intraepithelial lesions (LSIL) within the framework of the population-based cervical cancer screening program in Stockholm, Sweden. Samples were tested for HR-HPV E6/E7 mRNA by APTIMA (Gene-Probe Inc., San Diego, CA, USA). Women were followed up for 4 years after the index cytology via medical and laboratory records, and the Stockholm Oncology Center.

**Results:**

Nine of 25 (36%) women in the ASCUS group, and 64 of 180 (36%) women in the LSIL group developed cervical intraepithelial neoplasia (CIN) grade 2 or worse during 4 years of follow-up. 162 (74%) women were APTIMA-positive, and APTIMA had the highest sensitivity to predict CIN2 or worse and CIN3 or worse in the ASCUS (77.8% and 100%) and LSIL (78.1 and 75.8%) groups, although specificity was insufficient (<50%). HPV16 DNA testing and repeat cytology were more specific than APTIMA.

**Conclusion:**

The results of this population-based study with comprehensive follow-up support the use of APTIMA as a triage test for women with ASCUS. More focused investigation is required for women with LSIL.

## Introduction

Cytology-based cervical cancer screening programs have significantly reduced the incidence and mortality of cervical cancer [1], [2]. Most abnormalities detected in cytological screening are minor and non-specific. Cytology has a low positive predictive value (PPV) for the detection of cervical intraepithelial neoplasia (CIN) 2 or worse (CIN2+). There is also a high degree of inter-observer variability in cytological assessment, resulting in highly variable test accuracy [3]–[5].

Persistent infection with high-risk human papillomavirus (HR-HPV) is a prerequisite for developing precancerous cervical lesions and invasive cervical carcinoma (ICC) [6], [7]. Women infected with HPV16 and 18 are considered at particularly high risk and these types account for approximately 70% of ICC worldwide [8]. The relative risk of developing CIN2+ and CIN3 or worse (CIN3+) among HPV16-positive women compared to women positive for other HR-HPV types has been shown to be elevated (3.7 and 4.5, respectively) [9].

In Sweden today, approximately 8% of all cytological samples show some kind of abnormality, 80% of which are minor (i.e., atypical squamous cells of undetermined significance (ASCUS) or low-grade squamous intraepithelial lesions (LSIL)) [10]. According to national recommendations from 2010, women with minor cytological abnormalities should be referred for immediate colposcopy, with cervical biopsies or should be triaged with HPV DNA testing, preferable by reflex testing of a liquid-based cytology sample [11]. However, repeat cytology is still used as a follow-up method in some parts of Sweden.

Due to the high sensitivity (>90%) and negative predictive value (NPV) of HR-HPV DNA testing to predict CIN, HPV triage has become an attractive approach for the management of women with ASCUS [12]–[15]. However, HPV triage is not recommended in young women with LSIL, as the high prevalence of HR-HPV in this group leads to poor specificity for HPV testing [13], [14]. A test that maximizes sensitivity and specificity would allow more efficient and definitive triage.

HR-HPV infections results in progression to cervical cancer in only a small percentage of infected women, after a long period of latency. Thus, detection of mRNA transcripts of HPV genes known to be involved in oncogenesis may be more useful for detecting active and potentially persistent infection than HPV DNA tests. The expression of viral E6/E7 oncogenes of HR-HPV has been proposed as a marker of a transforming HPV infection and relevant clinical progression of cervical disease [16]–[18]. Up-regulation of these oncogenes triggers the degradation of p53 and retinoblastomaprotein, which, in turn,causes deregulation of the cell cycle, leading to malignant transformation [6]. Therefore, HR-HPV E6/E7 mRNA is a promising marker to predict the development of CIN2+ and ICC.

Studies of mRNA testing have shown consistently high sensitivity and a higher specificity than HR-HPV DNA testing both in primary screening and in ASCUS and LSIL triage [14], [19], [20]. Since APTIMA always detects full-length E6/E7 mRNA, a positive result should correlate very well with integrated HPV, loss of HPV replication, and stabilized E6/E7 full-length mRNA expression.

The present longitudinal study aims to compare, for the first time, the triage efficacy and usefulness of HR-HPV E6/E7 mRNA testing of APTIMA HPV Assay (APTIMA) to that of HPV16 DNA testing, HPV16/18 DNA testing, and repeat cytology in the population-based cervical cancer screening program.

## Patients and Methods

### Study Population

The study population was composed of 219 HR-HPV-positive women diagnosed with ASCUS or LSIL within the framework of the population-based cervical cancer screening program in Stockholm, Sweden. Details on recruitment have been described elsewhere [21]. Briefly, women with ASCUS or LSIL were referred for further investigation, including colposcopy, directed biopsies, and/or repeat cytology according to the screening program guidelines. Histological samples were evaluated and classified as within normal limits, CIN1, CIN2+ or CIN3+ based on the most severe lesion present [22]. Cytological results were classified according to the CIN classification of the Swedish Society for Clinical Cytology [22], but were re-classified using the Bethesda system for the purposes of this study, excluding koilocytosis without nuclear atypia from the LSIL diagnosis [23].

The mean age of the study participants was 32.0 (range: 23–60 years (standard deviation (SD) 8.5 years)). Half of the women were aged 30 years or younger and there was no statistically significant difference in age between the ASCUS and LSIL groups (p = 0.60). The mean age was 32.8 years (SD 9.0) and 31.9 years (SD 8.4) in the ASCUS and LSIL groups, respectively. The age distribution of the study participants was as follows: 25.1% were 23–24, 23.7% were 25–29, 15.1% were 30–34, 16.0% were 35–39, 10.5% were 40–44, and 9.6% were 45–60 years of age. Women were followed for 4 years after the index ASCUS/LSIL cytology, during which time all histological and cytological results were obtained through medical and laboratory records, and through the Stockholm Oncology Center in cases where the information were insufficient. Yearly follow-up of low-grade disease was performed according to local clinical recommendations. Treatment by conization was performed if low-grade disease persisted after two years or immediately if high-grade disease was diagnosed.

### HPV DNA Testing

HPV DNA testing had been previously performed on the baseline ASCUS/LSIL samples from this study population. HPV DNA was extracted (MagNA Pure LC robot, Roche Diagnostics, Pleasanton, California, USA) from a lysed cell pellet of 1 milliliter of the PreservCyt sample. The DNA of 37 HPV types [24] was detected and genotyped by the Linear Array Genotyping Test (Roche Diagnostics) according to the manufacturer’s instructions [21]. Beta-globin was included in the test as an internal control to test for sample adequacy and avoid false negatives.

### HPV E6/E7 mRNA Testing by APTIMA

In the present study, PreservCyt samples used for HPV DNA testing were retrieved from the archives and tested by APTIMA (Gene-Probe Inc., San Diego, CA, USA). Samples were transferred to 2.9 ml of buffered detergent solution and a 400 µl aliquot of the mixture was then tested according to the manufacturer’s instructions. APTIMA is a qualitative nucleic acid amplification test that detects the E6/E7 mRNA of 14 HR-HPV types (16, 18, 31, 33, 35, 39, 45, 51, 52, 56, 58, 59, 66 and 68), and has been validated for cervical specimens in PreservCyt medium [25], [26]. The test does not differentiate between HR-HPV types, and is designed not to cross-react with low-risk HPV6, 11, 42, 43, 44, or probable HR-HPV 53. An analytic cut-off of 1.00 was used to determine HPV interpretation. All laboratory analyses were performed by the Department of Virology, Karolinska Hospital [27].

### Statistical Analysis

The most severe histological or cytological diagnosis recorded during the 4-year follow-up was considered as the outcome. Accuracy parameters for the prediction of CIN2+ and CIN3+ were computed for APTIMA, HPV16 DNA testing, HPV16/18 DNA testing, and repeat cytology. The parameters calculated included: sensitivity, specificity, positive predictive value (PPV), and negative predictive value (NPV), diagnostic odds ratio and likelihood ratios (LR), stratified by ASCUS and LSIL diagnosis at baseline. Relative sensitivities, specificities, PPV, and NPV with 95% confidence intervals (CIs) were computed for APTIMA compared to HPV16 DNA testing, HPV16/18 DNA testing, and repeat cytology at three different cut-off levels : ASCUS+, LSIL+ and high-grade squamous intraepithelial lesions or worse (HSIL+).

Analyses were performed using Stata 13 (Stata, College Station, TX, USA).

### Ethics Statement

This study was approved by the Regional Ethical Review Board in Stockholm, Sweden (No. 04-679/3 and No. 2010/944-32) and written informed consent for all women were obtained before inclusion.

## Results

### HR-HPV DNA and APTIMA Results at Index Cytology

With regard to the distribution of the index smears, LSIL dominated (87.0%). All 219 women were HR-HPV DNA-positive and HPV16 was the most frequently detected HPV type in the ASCUS (20.7%) and LSIL groups (31.1%). The second most common HPV type in the ASCUS group was HPV53 (17.2%), while HPV51 and HPV52 were prevalent in the LSIL group (15.3%). HPV18 was found in 13.8% of the ASCUS and 11.6% of the LSIL group. In the ASCUS group, 31.0% were HPV16/18-positive as compared to 41.1% of the LSIL group. In total, 162 women (74.0%) were mRNA positive with the APTIMA-test, 17 (58.6%) from the ASCUS and 145 (76.3%) from the LSIL group (Table 1). The majority of HPV16/18-positive women were APTIMA-positive, regardless of index cytology.

**Table 1:** Type-specific HPV DNA distribution in the ASCUS and LSIL groups by APTIMA status.

	ASCUS group				LSIL group			
	APTIMA−		APTIMA+		APTIMA−		APTIMA+	
type	N	row %	N	row%	N	row %	N	row%
**16**	2	33.3%	4	66.7%	16	22.5%	55	77.5%
**18**	0	0.0%	4	100.0%	6	25.0%	18	75.0%
**31**	1	25.0%	3	75.0%	2	7.7%	24	92.3%
**33**	2	50.0%	2	50.0%	3	25.0%	9	75.0%
**35**	0	–	0	–	4	23.5%	13	76.5%
**39**	1	50.0%	1	50.0%	4	16.7%	20	83.3%
**45**	0	0.0%	1	100.0%	5	20.0%	20	80.0%
**51**	3	75.0%	1	25.0%	4	12.5%	28	87.5%
**52**	1	50.0%	1	50.0%	7	20.0%	28	80.0%
**56**	0	0.0%	3	100.0%	1	4.3%	22	95.7%
**58**	2	50.0%	2	50.0%	2	15.4%	11	84.6%
**59**	0	0.0%	1	100.0%	2	10.0%	18	90.0%
**66**	1	33.3%	2	66.7%	0	0.0%	18	100.0%
**68**	0	0.0%	2	100.0%	3	37.5%	5	62.5%
**26**	0	–	0	–	1	50.0%	1	50.0%
**53**	1	20.0%	4	80.0%	5	25.0%	15	75.0%
**73**	1	50.0%	1	50.0%	6	27.3%	16	72.7%
**82**	0	0.0%	3	100.0%	0	–	0	–
**16/18**	2	22.2%	7	77.8%	22	23.7%	71	76.3%
**Total**	**12**	**41.4%**	**17**	**58.6%**	**45**	**20.6%**	**145**	**79.4%**

HPV: human papillomavirus, ASCUS: atypical squamous cells of undetermined significance, LSIL: low-grade squamous intraepithelial lesions.

### Cytological and Histological Results during Follow-up

Results for repeat cytology within 12 months were the following: normal cytology was recorded in 113 (57.0%), ASCUS in 16 (8.1%), LSIL in 53 (26.3%), and atypical squamous cells-cannot rule out high-grade lesions (ASC-H) in 17 (8.6%). Histopathological results for 209 women during follow-up were missing in 8 (3.8%) women, not representative in 3 (1.4%), no CIN in 56 (26.8%), CIN1 in 69 (33.0%), CIN2 in 37 (17.7%), and CIN3+ in 36 (17.2%). Nine of 25 women in the ASCUS group (36.0%) and 64 of 180 (35.6%) women in the LSIL group developed CIN2+ during 4 years of follow-up.

Altogether, 205 women came for a follow-up visit within 12 months from the index cytology, whereof 198 had a repeat cytology and, if indicated by colposcopy, a biopsy was taken. Seven women had only a biopsy test result available. Four women came for follow-up visits later than 12 months and therefore their cytological test result could not be categorized as a repeat cytology. Ten women were lost to follow-up for unknown reasons. The characteristics of those that were lost to follow-up did not differ largely from the women that were followed, although the mean age was 30.3 years (slightly younger).

### Accuracy of Different Triage Options in the ASCUS Group

**Figure 1:**
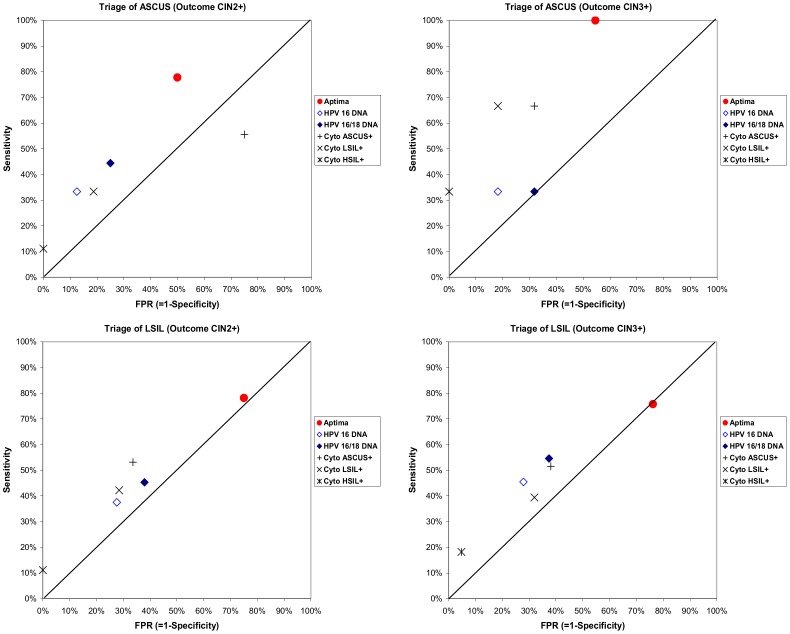
Sensitivity & FPR (False positive rate) of the different tests used to triage women with ASCUS (upper) or LSIL (lower) to detect CIN2+ (left) or CIN3+ (right). Red circle: APTIMA, rhombus without color HPV DNA 16, rhombus blue HPV16/18 DNA, cross: cytology AS-CUS as cut-off, diagonal cross: cytology with LSIL as cut-off, double diagonal cross: cytology with HSIL as cut-off. ASCUS-atypical squamous cells of undetermined significance, LSIL-low-grade squamous intraepithelial lesions, HSIL− high-grade squamous intraepithelial lesions. CIN− cervical intraepithelial neoplasia. HR-HPV: High-risk human papillomavirus.

The sensitivity of APTIMA to predict CIN2+ and CIN3+ was 77.8% (95% CI 40.0–90.0) and 100.0% (95% CI 40.0–100), respectively in the ASCUS group. Specificity to predict the absence of CIN2+ or CIN3+ was 50.0% (CI 30.0–70.0) and 45.5% (CI 30.0–60.0), respectively (Figure 1, Table 2).

**Table 2:** Overview of the sensitivity and specificity, PPV, NPV, the risk of disease^a^ in case of a negative test (cNPV-1-NPV), DOR and LR.

Triagegroup	Outcome	Test	Testcut-off	TP	FN	FP	TN	N	Sensitivity	Specificity	PPV	NPV	cNPV	DOR	LR+	LR–
ASCUS	CIN2+	APTIMA	+	7	2	8	8	25	77.8%	50.0%	46.7%	80.0%	20.0%	5.60	1.56	0.44
ASCUS	CIN2+	HPV16 DNA	+	3	6	2	14	25	33.3%	87.5%	60.0%	70.0%	30.0%	5.25	2.67	0.76
ASCUS	CIN2+	HPV16/18 DNA	+	4	5	4	12	25	44.4%	75.0%	50.0%	70.6%	29.4%	5.33	1.78	0.74
ASCUS	CIN2+	Repeat cytology	ASCUS+	5	4	12	4	25	55.6%	25.0%	29.4%	50.0%	50.0%	1.25	0.74	1.78
ASCUS	CIN2+	Repeat cytology	LSIL+	3	6	3	13	25	33.3%	81.3%	50.0%	68.4%	31.6%	4.33	1.78	0.82
ASCUS	CIN2+	Repeat cytology	HSIL+	1	8	0	16	25	11.1%	100.0%	100.0%	66.7%	33.3%	2.00	–	0.89
ASCUS	CIN3+	APTIMA	+	3	0	12	10	25	100.0%	45.5%	20.0%	100.0%	0.0%	2.50	1.83	0.00
ASCUS	CIN3+	HPV16 DNA	+	1	2	4	18	25	33.3%	81.8%	20.0%	90.0%	10.0%	3.00	1.83	0.81
ASCUS	CIN3+	HPV16/18 DNA	+	1	2	7	15	25	33.3%	68.2%	12.5%	88.2%	11.8%	1.67	1.05	0.98
ASCUS	CIN3+	Repeat cytology	ASCUS+	2	1	7	15	25	66.7%	68.2%	22.2%	93.8%	6.3%	3.75	2.10	0.49
ASCUS	CIN3+	Repeat cytology	LSIL+	2	1	4	18	25	66.7%	81.8%	33.3%	94.7%	5.3%	7.20	3.67	0.41
ASCUS	CIN3+	Repeat cytology	HSIL+	1	2	0	22	25	33.3%	100.0%	100.0%	91.7%	8.3%	11.00	–	0.67
LSIL	CIN2+	APTIMA	+	50	14	87	29	180	78.1%	25.0%	36.5%	67.4%	32.6%	14.36	1.04	0.88
LSIL	CIN2+	HPV16 DNA	+	24	40	32	84	180	37.5%	72.4%	42.9%	67.7%	32.3%	28.00	1.36	0.86
LSIL	CIN2+	HPV16/18 DNA	+	29	35	44	72	180	45.3%	62.1%	39.7%	67.3%	32.7%	26.43	1.19	0.88
LSIL	CIN2+	Repeat cytology	ASCUS+	34	30	39	77	180	53.1%	66.4%	46.6%	72.0%	28.0%	37.94	1.58	0.71
LSIL	CIN2+	Repeat cytology	LSIL+	27	37	33	83	180	42.2%	71.6%	45.0%	69.2%	30.8%	32.01	1.48	0.81
LSIL	CIN2+	Repeat cytology	HSIL+	10	54	3	113	180	15.6%	97.4%	76.9%	67.7%	32.3%	19.82	6.04	0.87
LSIL	CIN3+	APTIMA	+	25	8	112	35	180	75.8%	23.8%	18.2%	81.4%	18.6%	7.29	0.99	1.02
LSIL	CIN3+	HPV16 DNA	+	15	18	41	106	180	45.5%	72.1%	26.8%	85.5%	14.5%	26.95	1.63	0.76
LSIL	CIN3+	HPV16/18 DNA	+	18	15	55	92	180	54.5%	62.6%	24.7%	86.0%	14.0%	23.66	1.46	0.73
LSIL	CIN3+	Repeat cytology	ASCUS+	17	16	56	91	180	51.5%	61.9%	23.3%	85.0%	15.0%	21.49	1.35	0.78
LSIL	CIN3+	Repeat cytology	LSIL+	13	20	47	100	180	39.4%	68.0%	21.7%	83.3%	16.7%	19.40	1.23	0.89
LSIL	CIN3+	Repeat cytology	HSIL+	6	27	7	140	180	18.2%	95.2%	46.2%	83.8%	16.2%	24.71	3.82	0.86

PPV, positive predictive value, NPV: negative predictive value, DOR: diagnostic odds ratio, LR: likelihood ratio, TP: true positive, FN: false negative, FP: false positive, TN: true negative, N: number, ASCUS: atypical squamous cells of undetermined significance, CIN2+: cervical intraepithelial neoplasia grade 2 or worse, HPV: human papillomavirus, LSIL: low-grade squamous intraepithelial lesions, CIN3+: cervical intraepithelial neoplasia grade 3 or worse.

a he risks of disease cNPV = 1-NPV.

APTIMA was the most sensitive test for triage in the ASCUS group, but the difference reached statistical significance only when compared with repeat cytology using a cut-off of HSIL+ to predict CIN2+ (relative sensitivity 7.0, CI 1.1–45.9). APTIMA was significantly less specific than HPV16 DNA testing to predict CIN2+ and CIN3+ (relative specificity 0.6 (CI 0.3–0.9) and 0.6 (CI 0.3–0.9), respectively) and significantly less specific than repeat cytology using a cut-off of LSIL+ and HSIL+ to predict CIN3+ (0.6 (CI 0.3–0.9) and 0.4 (CI 0.3–0.7)) (Table 3).

**Table 3:** Relative sensitivity and specificity of APTIMA compared to other tests to triage women with ASCUS or LSIL for the outcomes CIN2+ or CIN3+^a^.

			Relative sensitivity			Relative specificity		
Triage group	Outcome	Test	Estimate	lower CIB	upper CIB	Estimate	lower CIB	upper CIB
ASCUS	CIN2+	HPV16 DNA	2.33	0.87	6.27	**0.57**	**0.34**	**0.96**
ASCUS	CIN2+	HPV6/18 DNA	1.75	0.78	3.93	0.67	0.38	1.17
ASCUS	CIN2+	Cyto at ASCUS+	1.40	0.71	2.77	2.00	0.75	5.33
ASCUS	CIN2+	Cyto at LSIL+	2.33	0.87	6.27	0.62	0.36	1.06
ASCUS	CIN2+	Cyto at HSIL+	**7.00**	**1.07**	**45.90**	0.50	0.31	0.82
ASCUS	CIN3+	HPV16 DNA	3.00	0.61	14.86	**0.56**	**0.34**	**0.91**
ASCUS	CIN3+	HPV16/18 DNA	3.00	0.61	14.86	0.67	0.39	1.14
ASCUS	CIN3+	Cyto at ASCUS+	1.50	0.67	3.34	0.67	0.39	1.14
ASCUS	CIN3+	Cyto at LSIL+	1.50	0.67	3.34	**0.56**	**0.34**	**0.91**
ASCUS	CIN3+	Cyto at HSIL+	3.00	0.61	14.86	**0.45**	**0.29**	**0.72**
LSIL	CIN2+	HPV16 DNA	**2.08**	**1.48**	**2.93**	**0.35**	**0.25**	**0.48**
LSIL	CIN2+	HPV16/18 DNA	**1.72**	**1.28**	**2.32**	**0.40**	**0.29**	**0.57**
LSIL	CIN2+	Cyto at ASCUS+	**1.47**	**1.13**	**1.92**	**0.38**	**0.27**	**0.53**
LSIL	CIN2+	Cyto at LSIL+	**1.85**	**1.35**	**2.54**	**0.35**	**0.25**	**0.49**
LSIL	CIN2+	Cyto at HSIL+	**5.00**	**2.79**	**8.97**	**0.26**	**0.19**	**0.35**
LSIL	CIN3+	HPV16 DNA	**1.67**	**1.09**	**2.54**	**0.33**	**0.24**	**0.45**
LSIL	CIN3+	HPV16/18 DNA	1.39	0.96	2.00	**0.38**	**0.28**	**0.52**
LSIL	CIN3+	Cyto at ASCUS+	**1.47**	**1.00**	**2.16**	**0.38**	**0.28**	**0.53**
LSIL	CIN3+	Cyto at LSIL+	**1.92**	**1.21**	**3.06**	**0.35**	**0.26**	**0.48**
LSIL	CIN3+	Cyto at HSIL+	**4.17**	**1.97**	**8.81**	**0.25**	**0.19**	**0.33**

HPV: human papillomavirus, ASCUS: typical squamous cells of undetermined significance, LSIL: low-grade squamous intraepithelial lesions, CIB: 95% confidence interval bound.

a Significant differences in bold.

The PPV for all test options ranged from 29.4–100.0% for CIN2+ and from 12.5–100.0% for CIN3+. The PPV of APTIMA (46.7% (95% CI 24.8–69.9) for CIN2+ and 20.0% (95% CI 7.0–45.2) for CIN3+ was lower than that of the other tests, with the exception of repeat cytology using a cut-off of ASCUS+ to predict CIN2+ (29.4%, 95% CI 13.3–53.1), and HPV 16/18 DNA testing to predict CIN3+ (12.5%, 95% CI 2.2–47.1), but the differences were not significant. The relative PPV of APTIMA compared to repeat cytology using a cut-off of HSIL+ was 0.5 (CI 0.3–0.8) to predict CIN2+ and 0.2 (CI 0.1–0.6) to predict CIN3+ (Table 4) which were significant.

**Table 4:** Relative risk of disease in case of a positive test (relative PPV) and in case of a negative test (relative cNPV) of APTIMA compared to other tests in the ASCUS and LSIL groups for the outcomes CIN2+ and CIN3+^a^.

			Relative PPV			Relative cNPV		
Triage group	Outcome	Test	Estimate	lower CIB	upper CIB	Estimate	lower CIB	upper CIB
ASCUS	CIN2+	HPV16 DNA	0.78	0.32	1.91	0.67	0.44	1.02
ASCUS	CIN2+	HPV16/18 DNA	0.93	0.39	2.25	0.68	0.44	1.05
ASCUS	CIN2+	Cyto at ASCUS+	1.59	0.64	3.96	0.40	0.19	0.85
ASCUS	CIN2+	Cyto at LSIL+	0.93	0.36	2.45	**0.63**	**0.41**	**0.98**
ASCUS	CIN2+	Cyto at HSIL+	**0.47**	**0.27**	**0.80**	**0.60**	**0.39**	**0.91**
ASCUS	CIN3+	HPV16 DNA	1.00	0.13	7.57	0.00	.	.
ASCUS	CIN3+	HPV16/18 DNA	1.60	0.20	12.99	0.00	.	.
ASCUS	CIN3+	Cyto at ASCUS+	0.90	0.18	4.40	0.00	.	.
ASCUS	CIN3+	Cyto at LSIL+	0.60	0.13	2.74	0.00	.	.
ASCUS	CIN3+	Cyto at HSIL+	**0.20**	**0.07**	**0.55**	0.00	.	.
LSIL	CIN2+	HPV16 DNA	0.85	0.59	1.24	1.01	0.79	1.28
LSIL	CIN2+	HPV16/18 DNA	0.92	0.64	1.32	1.00	0.78	1.27
LSIL	CIN2+	Cyto at ASCUS+	0.78	0.56	1.09	1.16	0.91	1.47
LSIL	CIN2+	Cyto at LSIL+	0.81	0.57	1.16	1.06	0.83	1.34
LSIL	CIN2+	Cyto at HSIL+	**0.47**	**0.33**	**0.69**	1.01	0.80	1.27
LSIL	CIN3+	HPV16 DNA	0.68	0.39	1.19	**1.28**	**1.09**	**1.50**
LSIL	CIN3+	HPV16/18 DNA	0.74	0.43	1.26	**1.33**	**1.13**	**1.56**
LSIL	CIN3+	Cyto at ASCUS+	0.78	0.45	1.35	**1.24**	**1.06**	**1.47**
LSIL	CIN3+	Cyto at LSIL+	0.84	0.46	1.53	1.12	0.95	1.31
LSIL	CIN3+	Cyto at HSIL+	**0.40**	**0.20**	**0.79**	1.15	0.98	1.35

HPV: human papillomavirus, ASCUS: typical squamous cells of undertermined significance, LSIL: low-grade squamous intraepithelial lesions, CIB: 95% confidence interval bound.

a Significant differences in bold.

The risk of disease following a negative triage test result (calculated as the complement of the NPV: cNPV = 1-NPV) ranged from 5.3–11.8% for the outcome CIN3+, with the exception of APTIMA, for which no risk was detected. A negative APTIMA test resulted in a lower risk of disease compared to the other tests, but the difference was only significant when compared to repeat cytology using a cut-off of LSIL+ and HSIL+ (relative cNPV 0.6, CI 0.4–0.9 and 0.6, CI 0.4–0.9, for LSIL+ and HSIL+ respectively).

### Accuracy of Different Triage Options in the LSIL Group

The sensitivity of APTIMA was 78.1% (95% CI 70.0–90.0 and 75.8% (95% CI 60.0–90.0) for predicting CIN2+ and CIN3+, respectively, in the LSIL group. The specificity for predicting the absence of CIN2+ and CIN3+ was 25.0% (95% CI 20.0–30.0) and 23.8% (95% CI 20.0–30.0), respectively (Table 2). APTIMA was significantly more sensitive for predicting CIN2+ and CIN3+ compared to all other tests, except HPV16/18 DNA testing to predict CIN3+, where the difference between APTIMA and HPV16/18 DNA testing was not significant (Table 3). However, APTIMA was significantly less specific compared to all the other tests.

PPVs ranged from 36.5–76.9% for CIN2+ and from 18.2–46.2% for CIN3+. The PPV of APTIMA was lower than the other tests, but this was only significant when compared with repeat cytology using a cut-off of HSIL+, where the relative PPV was 0.5 (CI 0.3–0.7) for CIN2+ and 0.4 (CI 0.2–0.8) for CIN3+.

The risk of disease was still high when triage tests were negative (cNPV ranged from 28.0–32.7% for CIN2+ and from 14.0–18.6% for CIN3+.). A negative APTIMA test did not result in or predict a decrease in the risk of disease compared to other tests, and the risk of CIN3+ was significantly higher for APTIMA negative women compared to negative HPV16 DNA, HPV16/18 DNA, or repeat cytology result using a cut-off of ASCUS+.

Most tests showed accuracy estimates that did not deviate strongly from the neutral diagonal line (LR+ and LR− near 1), indicating poor triage capacity (Figure 1).

## Discussion

HPV infection is a necessary factor in the etiology of ICC [6] and expression of the viral E6/E7 oncogenes is necessary for conversion to and maintenance of malignancy in cervical tissue. HPV testing is an excellent first screen to identify women with a higher risk of developing cervical cancer. However, as known HPV testing has only limited power to stratify low-grade from high-grade disease and can therefore not be used to efficiently triage patients further. Therefore, additional markers for triaging patients to avoid overtreatment and overlooking relevant lesions are needed. Potential triage markers tested in this study were HPV mRNA expression, HPV 16 DNA, HPV 16/18 DNA and repeated cytology.

In the current study, APTIMA detected 100% of CIN3+ and 77.8% of CIN2+ in the ASCUS group. APTIMA was the most sensitive test to predict high-grade CIN compared to HPV16 DNA testing, HPV16/18 DNA testing, and repeat cytology at three different cut-off levels. The specificity of APTIMA to exclude CIN2+ in the ASCUS group was 50.0%, and the PPV was 46.7%.

HPV DNA testing is widely accepted for ASCUS triage due to its higher sensitivity and similar specificity compared to repeat cytology (12), but newer assays like RNA-based APTIMA have also shown good performance in ASCUS triage (due to its higher specificity) [33]. Our results were similar to those of a meta-analysis of ASCUS triage, in which APTIMA maintained high sensitivity, but showed a greater specificity to detect cervical disease compared to Hybrid Capture 2 [33]. While the sensitivity of APTIMA was higher, in our study HPV16 DNA testing (specificity 87.5%) was significantly more specific. HPV 16 DNA testing identified women at the highest risk for cervical disease (PPV for CIN2+60.0%), but the sensitivity was low (33.3%). The 30% risk of disease, despite a negative HPV16 DNA result, indicated that these women cannot return to the normal screening schedule. Compared to HPV16 DNA testing, repeat cytology using a cut-off of LSIL+ yielded a low sensitivity, but an equally high specificity to detect high-grade CIN.

APTIMA-negative women in the ASCUS group had the lowest risk of disease, and no risk for CIN3+ was found. In the LSIL group, APTIMA was the most sensitive test, detecting 76% and 78% of all CIN3+ and CIN2+, respectively, but the least specific test (24–25% for detecting CIN3+ and CIN2+). Specificity for CIN3+ might not be considered useful in a clinical setting, as it would consider CIN2 results false-positives, which is not appropriate as most screening programs use CIN2 as the cut-off for treatment [28].

Our results are lower than the pooled sensitivity (91.0% and 96.7%) and specificity (42.5% and 38.7%) of APTIMA to detect CIN2+ and CIN3+ among LSILs reported in the aforementioned meta-analysis [33], which concluded that APTIMA might also be considered for LSIL triage. Other studies have also demonstrated difficulties with specificity in the LSIL group [29]. HPV DNA testing has not been recommended for LSIL triage because of its low specificity due to the high prevalence of HPV, especially in younger age groups. Fifty percent of our study women were under 30 years of age, which may have contributed to the observed low specificity, as lesions in young women may be more prone to regress. The use of CIN2+ as an outcome has also been an area of discussion, since the reproducibility of the diagnosis is considered poor [30]. A re-evaluation of the evidence for HPV66 [31] has revealed it to be a relatively common type, though it is rarely found in cancer, which could decrease the specificity and PPV of an assay that includes this type [31]. HPV66 is included in APTIMA, which may also have contributed to the low specificity.

A limitation of this study could be that specimens were stored for up to 5 years at room temperature in PreservCyt medium before APTIMA testing, as longer storage times might lead to mRNA degradation. However, two other studies have used PreservCyt specimens that had been stored for more than 3 years [29], [32] and concluded that mRNA was well preserved. We have investigated the use of APTIMA on samples from women undergoing testing for cervical cancer in a population-based routine screening program, reflecting a real-life setting and allowing us to apply our findings directly to routine clinical practice.

Strengths of the present study are the case verification and the data quality of the follow-up, based on unique personal identification numbers as part of the organized screening program and register via medical records and in cases with insufficient information data were supplemented with information from the Stockholm Oncology Center. The long observation time covers an entire 3-year screening interval which allows us to comment on the performance of the triage tests within the context of a programmatically relevant follow-up period.

Previous studies have compared the APTIMA test with the PreTect HPV-Proofer mRNA test (Norchip AS, Klokkarstua, Oslo, Norway) in women with ASCUS or LSIL cytology. The PreTect test detects mRNA of five HR-HPV types (16, 18, 31, 33, and 45). APTIMA was substantially more sensitive (ratio 1.91 (95% CI: 1.43–2.56) but less specific (ratio: 0.47 (95% CI: 0.34–0.63) for CIN2+) [33]–[35]. Rijkaart et al investigated whether HR-HPV mRNA detection by the PreTect HPV-Proofer can be used as a reflex test to stratify HR-HPV DNA-positive women of different cytological diagnoses for risk of CIN2+. The results showed that a positive PreTect HPV-Proofer reflex test conferred an increased risk of CIN2+ in HR-HPV DNA-positive women, particularly for those with normal cytology [36].

In summary, the tests evaluated showed accuracy estimates that indicated poor LSIL triage capacity, and the risk of disease remained even when triage tests were negative, indicating that these women cannot return to routine screening. In LSIL triage, our results suggested that, using a cut-off of ASCUS+, a negative HPV16 DNA, HPV16/18 DNA, or cytology resulted in lower risks of CIN over follow-up as compared to a negative APTIMA result. APTIMA showed only a limited ability to stratify the LSIL group according to disease risk, and therefore cannot be used to efficiently triage women with LSIL. Additional markers that can effectively triage these women and avoid over-treatment while not overlooking relevant lesions are needed. In the present study, APTIMA predicted 100% of CIN3+ and 77.8% of CIN2+ in the ASCUS group, making it the most sensitive test for detecting underlying high-grade cervical lesions. This corroborates existing data that APTIMA is an excellent test to identify those women with ASCUS who have a higher risk of developing high grade lesions and ICC.
